# A new surgical treatment for abdominal wall defects: A vascularized ribs-pleural transfer technique that can be used with or without a thoracic umbilical flap a case report

**DOI:** 10.1097/MD.0000000000009993

**Published:** 2018-03-02

**Authors:** Qiang Chen, Qi Liu, Yan Suo, Qingping Xie

**Affiliations:** Department of Hand Surgery, Zhejiang Provincial People's Hospital, People's Hospital of Hangzhou Medical College, Hangzhou, Zhejiang, China.

**Keywords:** abdominal wall defect, ribs-pleural transfer, vascularized

## Abstract

**Rationale:**

Abdominal wall defects are common after tumor resection.

**Patient concerns:**

We report an 83-year-old male patient with recurrent tumors in his abdomen, and who had an incision wound that could not be directly closed. Mesh was not suitable because the wound was infected.

**Diagnoses:**

Abdominal wall defect result from the resection of recurrent tumor.

**Interventions:**

We carried out a vascularized ribs-pleural transfer operation.

**Outcomes:**

After the surgery, the patient gained a functional recovery. No evidence of recurrence was noted 1 year after operation, and the patient showed no symptoms of abdominal compression syndrome.

**Lessons:**

We discuss the clinical diagnosis, treatment, and follow up and argue that the vascularized ribs-pleural transfer technique is a good method to deal with abdominal wall defects.

## 1 Introduction

In clinical work, it is common to see defective abdominal walls resulting from tumor resection, because surgical work involving the abdominal wall can be associated with infection, dehiscence, compartment syndrome, and other associated problems. The use of synthetic, nonabsorbable mesh is ideally suited for fascial defects in clean wounds with sufficient skin and subcutaneous tissue.^[1]^ However, mesh is not suitable for infected wounds, and, previously, no method was proven suitable for treating infected wounds. Here we report a new vascularized ribs-pleural transfer technique—which can be used with or without a thoracic umbilical flap—designed to repair upper abdominal wall defects. Flap reconstruction can be performed at the same time as transversalis fascia reconstruction and offers immediate and definitive wound closure. We report a case of an 83-year-old male with a large hernia resulting from tumor resection, as well as his functional recovery following the vascularized ribs-pleural transfer. To date no articles reporting this kind of treatment have referred to the same patient.

## 2 Case presentation

**Figure 1:**
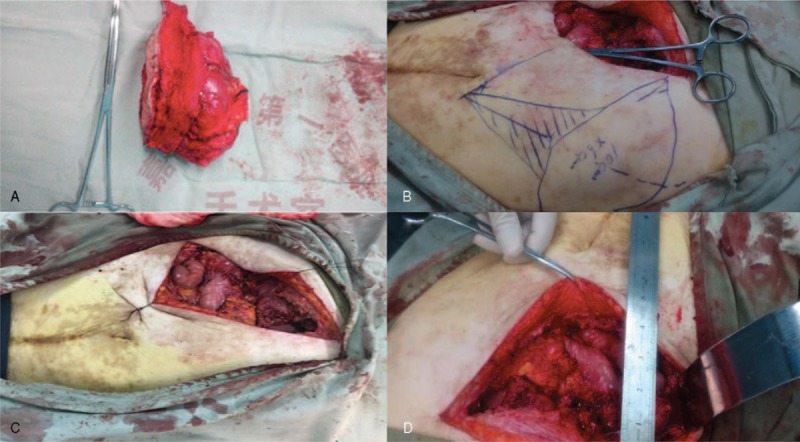
(A) Abdominal soft tissue tumor resection; (B) design of vascularized partial 9 to 10 ribs-pleural transfer technique without an umbilical flap; (C) abdominal wall full- thickness defect; (D) defect approximately 15 × 8 cm in size.

**Figure 2:**
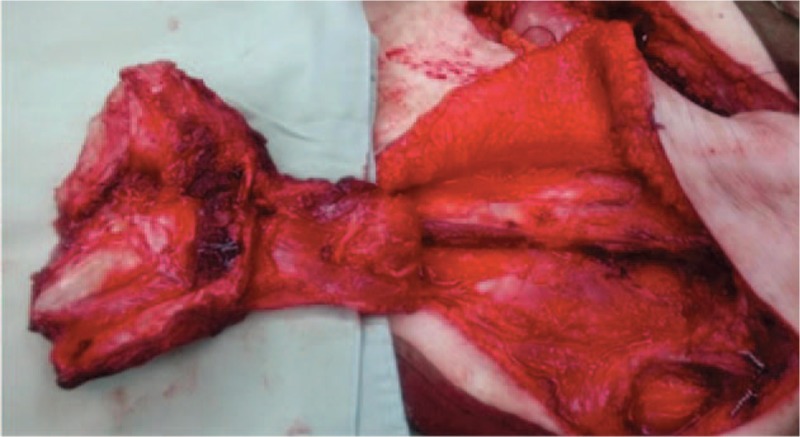
The vascularized partial ribs-pleural transfer technique after harvest. This was almost free, and was only connected by the artery pedicle.

An 83-year-old male patient came to the Department of Hand and Reconstructive Surgery of the People's Hospital (Zhejiang Province, China) complaining of mild pain and swelling, and upon inspection a mass was found in his left upper abdomen. This mass had been slowly increasing in size for 2 years. Two years ago, the patient had undergone an operation to remove an abdominal soft tissue fibrosarcoma. The local general surgeon had used a mesh to repair the peritoneal defect and to close the abdominal wall directly. Unfortunately, the incision did not heal due to infection, and the mesh was soon taken out by operation. The incision resulted in a draining wound, and bacterial cultures eventually showed that the wound had a methicillin-resistant *Staphylococcus aureus* infection. The patient repeatedly came to the clinic to have his bandage replaced, but the masses in his abdomen gradually increased in size. The patient was referred to the Hand Department, and while laboratory tests were normal, further examination by computed tomography imaging revealed that the tumor had recurred, and that the patient also had a hernia in the upper abdomen along with the infected wound. Considering the patient's condition, the main purpose of the operation was to resect the tumor and treat the hernia. The surgical procedure was designed to resect the recurrent tumor using a left upper abdomen approach under general anesthesia, but a very large (15 × 8 cm) abdominal wall defect was found on peritoneum, which could not be directly sutured (Fig. 1). In this case there was no obvious skin defect. Because of this defect, a right upper abdominal lateral approach for the operation was planned. The operation was planned to include a vascularized partial 9 to 10 ribs-pleural transfer (without a thoracic umbilical flap) by the vessel pedicle of the deep inferior epigastric artery (DIEA). The vascularized partial 9 to 10 ribs-pleural transfer material was first harvested so that it was connected only by the artery pedicle (Fig. 2).

**Figure 3:**
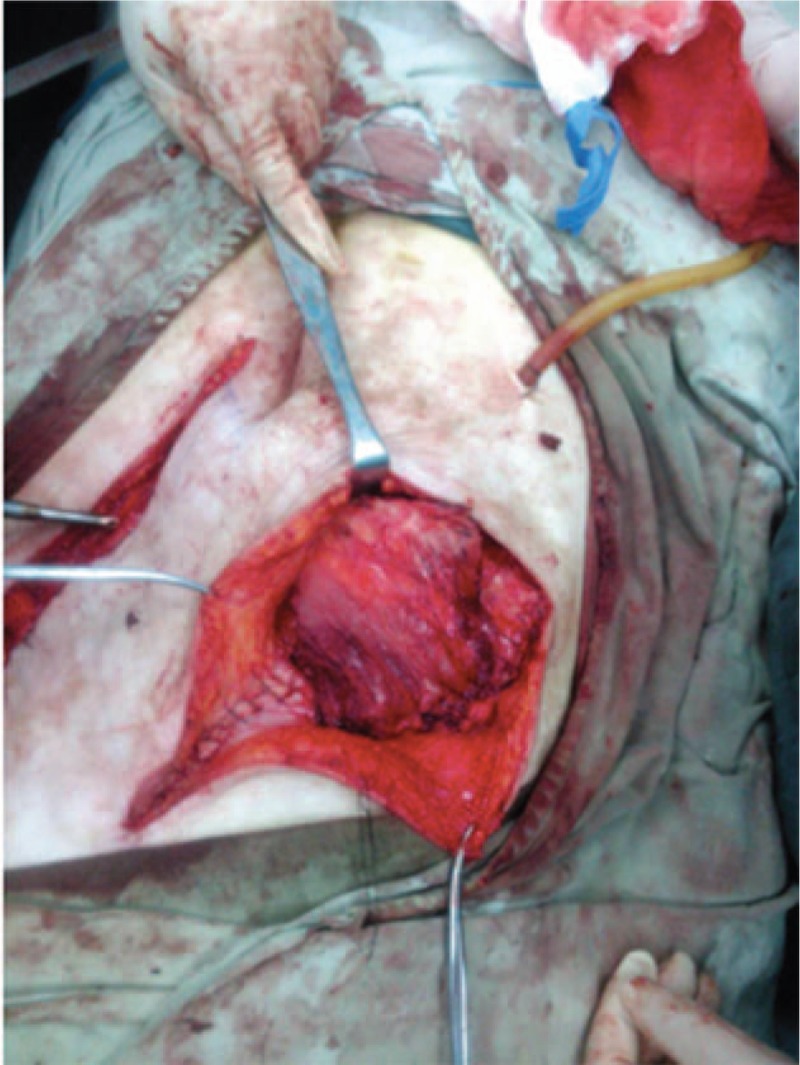
The vascularized partial 9 to 10 ribs-pleural transfer without a thoracic umbilical flap was turned so that the left abdominal wall defect recipient site would fill in the defect of the peritoneum.

The vascularized partial 9 to 10 ribs-pleural transfer was translocated to the left abdominal wall defect site via subcutaneous tunnel. The composite tissue was used to fill in the abdominal wall defect (Fig. 3). First, we sutured the broken peritoneum to the pleura, and then sutured the intercostal muscles to the abdominal muscles. Next, we closed the skin and the abdominal wall defect. Examinations after the surgery revealed that the right lung was not harmed. The right rib donor was wired closed using 1 mm steel wire, and the muscles, subcutaneous tissue, and skin were all sutured directly.

**Figure 4:**
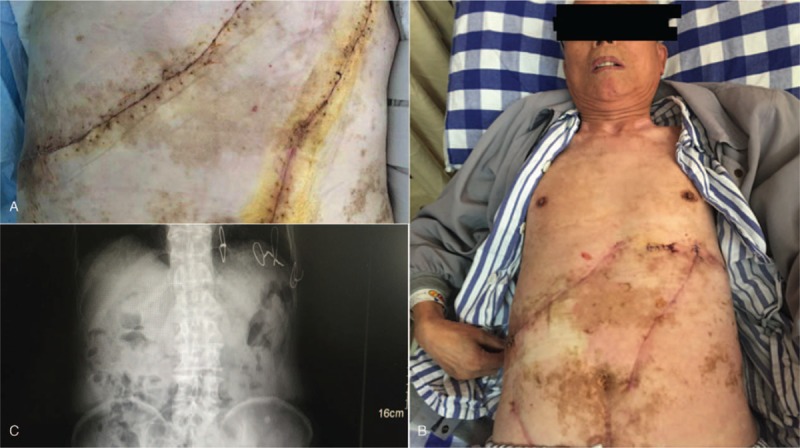
(A and B) Postoperative follow-up 3 months after surgery showed good healing; (C) X-ray radiography shows that the ribs were healing well.

Three days after the surgery, the patient's basal temperature returned to normal and he began to eat a full liquid diet. Six days after the surgery, we pulled out a subcutaneous drainage tube and the patient was able to eat half liquid diet. The patient had been given an infusion of Claforan for 1 week (4 g every 12 h; North China Pharmaceutical Co. Ltd, China). Ten days after the surgery, we removed the abdominal cavity drainage tube and the patient had recovered to the point where he could eat a normal diet. Two weeks after the surgery the incision healed, sutures were removed, and the patient was discharged. One year after the surgery, no masses in his abdomen were evident, and magnetic resonance imaging scans revealed no evidence of tumor recurrence. The patient also reported no abdominal pain or swelling symptoms. The patients’ diet and breathing were normal and the patient was then able to be able to resume his daily activities. Nevertheless, we continued to observe and follow up with the patient (Fig. 4).

These study protocols were approved by the medical ethics committee of the Zhejiang Provincial People's Hospital. Written informed consent for publication of clinical details and images was obtained from the patient. A copy of the consent form is available for review upon request.

## 3 Discussion

Significant soft-tissue loss in the abdominal wall implies the inability to recruit local tissue to close the abdominal wall defect. However, any effort to close the abdomen with tension invites infection, dehiscence, and associated problems. Multiple clinical problems can lead to a loss of abdominal wall soft-tissue and thereby require flap reconstruction; these problems include traumatic injury, soft tissue infection, and common oncological resections. Ideally, surgery should involve treatment by suturing without tension.^[1]^

Abdominal wall defects can be classified as partial-thickness or full-thickness defects. Partial-thickness defects involve the skin and subcutaneous tissue only, while full-thickness defects involve loss of the abdominal wall peritoneum as well as overlying skin, subcutaneous tissue, and transversalis fascia. Mesh is commonly used as a material for repairing abdominal wall defects, because it is able to avoid adhesion. However, complications of prosthetics include infection, seroma, extrusion, fistula, and hernia.^[2]^ In addition to repairing skin and subcutaneous tissues, repairing muscular components—especially the linea alba—is ultimately vital structure for abdominal wall reconstruction.^[3]^ The use of synthetic, nonabsorbable mesh is ideally suited for fascial defects in clean wounds with sufficient skin and subcutaneous tissue, but mesh cannot be applied if the wound is infected. In many patients, such as the patient in the case reported in this article, the mesh had to be removed due to infection. To repair full-thickness abdominal wall defects in the upper abdomen, a vascularized ribs-pleural transfer could be designed either with or without an umbilical skin flap, depending on whether a large skin defect was present. In this case, the peritoneal defect was repaired using a vascularized ribs-pleural transfer, and the skin and subcutaneous tissues were then repaired using an umbilical skin flap. The ribs-pleural transfer and flap both were both maintained with a sufficient supply of blood by the artery of the DIEA pedicle. Because this flap was a region flap, it did not require vascular anastomosis to have a stable blood supply. In principle, it would also be possible to anatomize the artery of the DIEA to obtain a long pedicle, so then composite tissue could be transferred to any place in the abdomen.^[4–7]^

Meruta et al reported a full-thickness thoracic wall reconstruction after tumor resection by rectus abdominis.^[8]^

An abdominal flap is commonly used for breast reconstruction, although there are many potential pitfalls that must be avoided to achieve a successful outcome.^[9]^ Bittar et al used a transverse rectus abdominis myocutaneous (TRAM) flap for breast reconstruction. The primary abdominal wound closure included fascial closure with or without reinforcing mesh.^[10]^ Elkwood et al used a pedicled TRAM flap in conjunction with a bony anchoring reinforcement system for abdominal wall reconstruction.^[11]^ Houdek et al reported a vascularized sole rib transplant for bone reconstruction in the defect of humerus shaft,^[12]^ and Jang et al reported using a vascularized costochondral bone graft for condyle and infratemporal fossa reconstruction.^[13]^

None of these studies mentioned peritoneal reconstruction. Our method was used on a patient who had not been the subject of any prior studies. In our opinion, our procedure is a good method to repair full-thickness defects in the abdominal wall.

## 4 Conclusion

This case report reports that, as a result of the positive clinical outcomes of our patient's treatment, we believe that the vascularized ribs-pleural transfer technique reported here is a good method to repair abdominal wall defects, including abdominal hernias. After operation, the patient can reach a desirable result and functional recovery.

## References

[1] Pushpakumar SB Wilhelmi BJ Van-Aalst VC Jr Abdominal wall reconstruction in a trauma setting. Eur J Trauma Emerg Surg 2007;33:3–13. 26815969 10.1007/s00068-007-7023-7

[2] Baumann DP Butler CE Flap Reconstruction of the Abdominal Wall. New York, NY: Springer International Publishing; 2016.

[3] Majumder A Clinical Anatomy and Physiology of the Abdominal Wall. New York, NY: Springer International Publishing; 2016.

[4] Zheng L Dong ZG Zheng J Deep inferior epigastric vessel-pedicled, muscle-sparing rectus abdominis myocutaneous (RAM) flap for reconstruction of soft tissue defects in pelvic area. Eur J Orthop Surg Traumatol 2015;25:859–63. 25633126 10.1007/s00590-015-1599-0

[5] Hou C Chang S Lin J Springer International Publishing, Surgical Atlas of Perforator Flaps, 2015;18 1:131–133.

[6] Mori H Akita K Hata Y Anatomical study of innervated transverse rectus abdominis musculocutaneous and deep inferior epigastric perforator flaps. Surg Radiol Anat 2007;29:149–54. 17318283 10.1007/s00276-007-0187-3

[7] Ahmad Z Sadideen H Oliver C The vertical pedicled DIEP flap: an alternative for large perineal reconstructions after tumour excision. Eur J Plast Surg 2015;38:331–4.

[8] Meruta AC Quoc CH Toussoun G Full thickness thoracic wall reconstruction after oncologic surgery. Eur J Plast Surg 2013;36:495–502.

[9] Miyagi K Candia MD Patel AJK Avoiding Pitfalls in Microvascular Breast Reconstruction. New York, NY: Springer International Publishing; 2016.

[10] Bittar S Ostric SA Martin WJ Abdominal closure for a free transverse rectus abdominis myocutaneous flap: macrosurgery's importance in microsurgery. Aesthetic Plast Surg 2006;30:253–4. 16547637 10.1007/s00266-005-0144-7

[11] Elkwood AI Ashinoff RL Kaufman MR Using pedicled TRAM flap in conjunction with the bony anchoring reinforcement system (BARS) for abdominal wall reconstruction. Eur J Plast Surg 2014;37:381–6.

[12] Houdek MT Wagner ER Wyles CC New options for vascularized bone reconstruction in the upper extremity. Semin Plast Surg 2015;29:20–9. 25685100 10.1055/s-0035-1544167 PMC4317278

[13] Jang HW Kim NK Lee WS Mandibular condyle and infratemporal fossa reconstruction using vascularized costochondral and calvarial bone grafts. J Korean Assoc Oral Maxillofac Surg 2014;40:83–6. 24868505 10.5125/jkaoms.2014.40.2.83 PMC4028791

